# Multiplex quantification of C-terminal alpha-1-antitrypsin peptides provides a novel approach for characterizing systemic inflammation

**DOI:** 10.1038/s41598-022-07752-w

**Published:** 2022-03-09

**Authors:** Arite Bigalke, Christoph Sponholz, Claudia Schnabel, Michael Bauer, Michael Kiehntopf

**Affiliations:** 1 Department of Clinical Chemistry and Laboratory Medicine, Jena University Hospital, Jena, Germany; 2 Department of Anesthesiology and Intensive Care Therapy, Jena University Hospital, Jena, Germany

**Keywords:** Biomarkers, Diseases, Molecular medicine

## Abstract

C-terminal peptides (CAAPs) of the highly abundant serine protease alpha-1-antitrypsin (A1AT) have been identified at various lengths in several human materials and have been proposed to serve as putative biomarkers for a variety of diseases. CAAPs are enzymatically formed and these enzymatic activities are often associated with excessive immune responses (*e.g.* sepsis, allergies). However, most of those CAAPs have been either detected using in vitro incubation experiments or in human materials which are not easily accessible. To gain a comprehensive understanding about the occurrence and function of CAAPs in health and disease, a LC–MS/MS method for the simultaneous detection of nine CAAPs was developed and validated for human plasma (EDTA and lithium-heparin) and serum. Using this newly developed method, we were able to detect and quantify five CAAPs in healthy individuals thereby providing an initial proof for the presence of C36, C37, C40 and C44 in human blood. Concentrations of four CAAPs in a clinical test cohort of patients suffering from sepsis were significantly higher compared to healthy controls. These results reveal that in addition to C42 other fragments of A1AT seem to play a crucial role during systemic infections. The proposed workflow is simple, rapid and robust; thus this method could be used as diagnostic tool in routine clinical chemistry as well as for research applications for elucidating the diagnostic potential of CAAPs in numerous diseases. To this end, we also provide an overview about the current state of knowledge for CAAPs identified in vitro and in vivo.

## Introduction

The endogenous balance between inhibitory active forms of the major human SERPIN alpha-1-antitrypsin (A1AT) and its target-proteinases (primarily human neutrophil elastase, HNE) is a crucial factor determining whether local inflammation processes will result in larger connective tissue damage^1–3^. Complex formation of A1AT with serine proteases does not only cause inactivation of both substrates but also cleavage of the SERPIN at the reactive site centered at Met^382^-Ser. However, several non-target enzymes are also able to cleave A1AT in the RCL (reactive center loop) region causing functional loss of the primary regulator towards human serine proteinases and thus increase the risk for the development of lung emphysema and other acute or chronic inflammatory conditions which are known pathophysiological consequences among A1AT deficient individuals^4^.

During the process of A1AT cleavage by certain enzymes (with or without complex formation), carboxyl-terminal peptides of A1AT (CAAPs) of various lengths can emerge. In addition to HNE which provokes the formation of C36 when complexed to A1AT^5^, many other enzymes have been identified exhibiting efficient A1AT degradation activity with simultaneous generation of CAAPs. Those enzymes include either endogenous human proteases (mainly matrix-metalloproteinases)^5–25^, proteases found in microbes causing infections^26–32^ or proteases that act as allergens for human allergic disorders^33–35^. However, CAAPs have been detected predominantly by using in vitro incubation experiments with purified A1AT. A few CAAPs have also been identified in vivo in human tissue and liquids such as urine^36^, bronchoalveolar lavage fluid^37,38^, gingival crevicular fluid^39^, spleen and bile^40^, lung tissue^41^, carotid artery tissue^42^, placenta^43,44^ and nipple aspiration fluids^45^. Some CAAPs were proposed to serve as putative biomarkers for glomerular kidney diseases^36^, pulmonary fibrosis^38^, gingivitis^39^ and carotid artery stenosis^42^. However, C42 (“CAAP48/47”) is the hitherto only CAAP detected in human blood^46^. In this previous study, C42 has been shown to function as inflammatory marker because C42 levels were significantly elevated in patients with severe sepsis compared with patients with systemic inflammatory response syndrome (SIRS) or HIV, respectively. Due to the higher concentrations of C42 found in the sepsis compared to SIRS group, a crucial role of microbes frequently triggering septic shock can be assumed. This is supported by the fact that extracellular microbial proteases, identified in vitro as CAAP-generating enzymes, serve as virulence factors for *Staphylococcus aureus *^27–29^, *Pseudomonas aeruginosa *^27,30^, *Serratia marcescens*^27,31^ and *Candida albicans *^47^. In addition to the role of C42 as sepsis biomarker, CAAPs reveal immunomodulatory functions as well and thus might contribute themselves to the inflammatory process. Among the multiplicity of A1AT cleavage fragments, C36 and C42 are the best studied CAAPs regarding their inflammatory immune modulating functions. They act, for example, as chemoattractant for phagocytes and monocytes^48^, activators of human monocytes and neutrophils in vitro^41,42,46,49^, neutrophil extracellular trap inhibitory substances^50^ and alter hepatic functions and gene expression in vitro and in vivo^51,52^. From a pathophysiological perspective, CAAPs seem to be highly relevant as diagnostic tools for the identification of a variety of diseases. However, the diagnostic use of CAAP profiles is still associated with some challenges. First, CAAPs are likely not equally distributed among the human body. Second, many enzymes are capable to cleave A1AT at more than only one position in the RCL region. Third, knowledge gained from in vitro experiments cannot directly translated to natural and therefore more complex environments such as the human body.

To better understand the complexity of mechanisms leading to CAAP formation, we focused on the development of a diagnostic tool determining CAAP patterns. To this end, we implemented a fast, multiplexed LC–MS/MS method for the simultaneous quantification of nine CAAPs in human serum and plasma; readily available and widely-used biomaterial.

## Results

A variety of cleavage products of the major plasma protease inhibitor alpha-1-antitrypsin (CAAPs) have been observed in vitro by incubation of alpha-1-antitrypsin with proteases as well as in vivo in several human tissues. With the exception of C42 (“CAAP48/47”)^46^, none of those CAAPs have been detected in human blood yet. Due to the vital role of CAAPs during inflammatory infection and possibly other diseases, a rapid method using readily available biomaterials such as blood-derived specimens is needed. Therefore, nine full-length C-terminal peptides were selected and synthesized compromising each CAAP amino acid lengths (from C45 to C22) in WT (wild type) and SNP (single nucleotide polymorphism) variant rs1303^53^, respectively. In order to be able to quantitatively measure this peptide panel a multiplex MRM-based method was established and validated^54,55^ for human plasma (EDTA and lithium-heparin) and serum.

## Method validation

### Linearity, working range, carry-over and specificity

Nine calibrants were used in total ranging from 0.01 µM to 1.5 µM thereby covering more than two orders of magnitude in concentration. In eight independent analytical runs, mean coefficients of determination of each calibration curve from 0.01 to 1.5 µM were R^2^ ≥ 0.996 for each compound (CV < 0.4%, n = 8, respectively) except for C43^WT^ and C43^SNP^ which were R^2^ ≥ 0.992 (CV ≤ 0.31%, n = 8, respectively). These results demonstrate sufficient *linearity* throughout the entire calibration range. Working ranges for each of the CAAPs were set according to concentrations in healthy and critically ill patients and were optimized during validation procedure (Table 1).

**Table 1:** Working ranges, QC sample concentrations and results of carry-over experiments. Carry-over is determined as the ratio of peak areas measured in blank albumin sample to peak areas of respective LLOQs (%).

Analyte	Working range (no. of calibrants)	Concentrations of QCs used for validation	Carry-over
C36^WT^	0.025–1.5 µM (8)	QC2 (0.025 µM, LLOQ)/ QC3 (0.3 µM)/ QC4 (0.9 µM)	4%
C36^SNP^			2%
C42^WT^			2%
C42^SNP^			1%
C37^WT^	0.01–1 µM (8)	QC1 (0.01 µM, LLOQ) QC3 (0.3 µM) QC4 (0.9 µM)	0%
C37^SNP^			0%
C22^WT^	0.01–0.5 µM (7)	QC1 (0.01 µM, LLOQ) QC2 (0.025 µM) QC3 (0.3 µM)	0%
C22^SNP^			1%
C39^WT^			0%
C39^SNP^			0%
C40^WT^			0%
C43^WT^			0%
C43^SNP^			2%
C44^WT^			0%
C44^SNP^			2%
C45^WT^			0%
C45^SNP^			0%
C40^SNP^	0.0036–0.1785 µM (7)	QC1 (0.0036 µM, LLOQ) QC2 (0.089 µM) QC3 (0.1073 µM)	0%

*Carry-over was* less than 5% of peak areas compared to respective LLOQ samples for all CAAPs and are thus in line with official recommendations (below 20% for analytes, Table 1)^55^. In none of the blank albumin samples measured directly after respective ULOQ samples internal standards were detected.

No interferences (test of *specificity*) above the LLOQ were detected (Additional file 2). We detected a few WT-variants above 20% of the LLOQ in the presence of respective SNP-variants. There was further no interference greater than 5% between CAAPs and internal standard detection (Additional file 2).

### Accuracy and precision

Mean accuracies and precisions (within-run repeatability and between-run) were within recommended ranges (maximum ± 15% and ± 20% for LLOQ, respectively) (Table 2).

**Table 2:** Accuracy and precision of repeated measurements.

Analyte	Quality control	Within-run repeatability (technical replicates, n = 5)		Between-run (independent runs, n = 5)	
		Accuracy (mean, %)	Precision (mean CV, %)	Accuracy (mean, %)	Precision (mean CV, %)
C36^WT^	QC2/ QC3/ QC4	110/ 111/ 107	5/ 2/ 1	113/ 108/ 106	2/ 3/ 2
C36^SNP^		113/ 108/ 104	1/ 3/ 1	113/ 107/ 104	1/ 2/ 2
C42^WT^		109/ 107/ 104	2/ 2/ 3	110/ 106/ 105	3/ 2/ 3
C42^SNP^		107/ 106/ 104	3/ 2/ 3	109/ 106/ 105	2/ 2/ 3
C37^WT^	QC1/ QC3/ QC4	117/ 108/ 104	4/ 2/ 2	121/ 108/ 104	6/ 3/ 2
C37^SNP^		113/ 107/ 105	5/ 2/ 3	117/ 105/ 103	2/ 2/ 3
C22^WT^	QC1/ QC2/ QC3	99/ 100/ 100	2/ 3/ 3	103/ 105/ 102	4/ 4/ 2
C22^SNP^		98/ 101/ 102	3/ 2 / 4	101/ 105/ 102	7/ 3/ 3
C39^WT^		120/ 114/ 109	5/ 1/ 1	117/ 119/ 106	2/ 4/ 4
C39^SNP^		111/ 111/ 103	3/ 4/ 2	117/ 110/ 103	7/ 3/ 1
C40^WT^		115/ 112/ 106	3/ 4/ 2	117/ 112/ 106	2/ 3/ 2
C40^SNP^		112/ 108/ 104	8/ 6/ 2	105/ 103/ 101	5/ 2/ 2
C43^WT^		110/ 108/ 99	3/ 2/ 2	117/ 111/ 101	4/ 4/ 2
C43^SNP^		109/ 107/ 100	5/ 2/ 2	117/ 112/ 103	5/ 3/ 3
C44^WT^		117/ 113/ 103	1/ 2/ 2	118/ 112/ 103	10/ 7/ 4
C44^SNP^		112/ 107/ 103	2/ 2/ 2	113/ 111/ 102	9/ 9/ 4
C45^WT^		117/ 116/ 104	2/ 2/ 2	115/ 111/ 104	8/ 8/ 4
C45^SNP^		112/ 111/ 102	5/ 1/ 2	111/ 111/ 102	6/ 5 /4

### Selectivity and investigation of matrix effects

Selectivity was determined in blank albumin samples and results revealed that no peaks (analyte or internal standards) were detected. Serial dilutions of CAAPs standard in pool (human) matrices verified that the responses of concentrations in albumin, serum and plasma (EDTA and lithium-heparin) are linear, respectively (Additional file 3). Due to the unavailability of analyte-free biological matrix, matrix effects were investigated by determining recovery rates and precisions of each compound in different human whole blood derived specimens which were compared to zero albumin samples with equal amount of CAAPs added (using area ratios instead of back-calculated concentrations). Most of the analytes spiked into EDTA plasma were within ± 20% of recovery compared to zero albumin sample (Table 3). Precisions were ≤ 10% between six individually analyzed human donors suggesting that matrix effects are low in EDTA plasma. Recovery rates of CAAPs in lithium-heparin as well as in serum were clearly lower than those in EDTA plasma revealing that effects of these matrices are considerably high. However, most of the CAAP concentrations in serum were consistently back-calculated among the six measured healthy individuals (precision ≤ 20%).

**Table 3:** Recovery rates of spiked CAAP concentrations in different biological matrices. Accuracies and precisions are shown as mean of six voluntary donors. H: high CAAPs concentration; L: low CAAPs concentration.

	EDTA plasma				Lithium-heparin plasma				Serum			
	Accuracy (mean, n = 6)		Precision (n = 6)		Accuracy (mean, n = 6)		Precision (n = 6)		Accuracy (mean, n = 6)		Precision (n = 6)	
	H	L	H	L	H	L	H	L	H	L	H	L
C22^WT^	98%	99%	4%	1%	46%	46%	10%	13%	58%	59%	8%	13%
C22^SNP^	98%	99%	3%	2%	46%	46%	11%	13%	58%	59%	8%	14%
C36^WT^	99%	91%	2%	5%	25%	20%	21%	68%	49%	50%	10%	18%
C36^SNP^	105%	109%	4%	4%	28%	29%	24%	38%	52%	53%	12%	14%
C37^WT^	125%	126%	5%	6%	40%	42%	14%	14%	66%	65%	5%	9%
C37^SNP^	129%	132%	4%	3%	51%	55%	11%	17%	80%	80%	6%	9%
C39^WT^	84%	78%	6%	8%	31%	33%	16%	21%	47%	44%	11%	17%
C39^SNP^	83%	80%	9%	10%	19%	21%	25%	30%	30%	29%	21%	27%
C40^WT^	117%	105%	5%	8%	28%	28%	19%	22%	47%	43%	12%	14%
C40^SNP^	102%	102%	5%	2%	27%	29%	17%	21%	43%	44%	13%	17%
C42^WT^	100%	97%	2%	7%	36%	34%	13%	31%	52%	52%	9%	17%
C42^SNP^	99%	103%	4%	7%	28%	28%	21%	31%	39%	39%	17%	21%
C43^WT^	119%	121%	5%	6%	44%	45%	12%	13%	57%	55%	5%	7%
C43^SNP^	113%	116%	3%	6%	33%	34%	14%	15%	38%	40%	11%	12%
C44^WT^	81%	79%	8%	5%	18%	18%	19%	28%	25%	23%	11%	20%
C44^SNP^	82%	80%	8%	6%	15%	17%	25%	39%	21%	22%	15%	23%
C45^WT^	77%	72%	7%	10%	24%	20%	19%	23%	35%	27%	7%	14%
C45^SNP^	88%	88%	8%	5%	20%	24%	21%	22%	30%	30%	12%	16%

To clarify reasons for high variations of peak area ratios of CAAPs in lithium-heparin plasma, variations of internal standards were additionally analyzed (Table 4).

**Table 4:** Mean peak areas and precisions of internal standards in different biological matrices. Different CAAP concentrations were spiked into each of the individual matrices: 0 = pure albumin; H: high CAAPs concentration; L: low CAAPs concentration.

	No. of values	Peak Area					
		C42^IS^		C37^IS^		C22^IS^	
		mean	CV (%)	mean	CV (%)	mean	CV (%)
Albumin (H, L)	n = 4	9.2*10^6^	2.7%	7.0*10^6^	3.0%	7.9*10^6^	1.2%
EDTA (0, H, L)	n = 34	7.7*10^6^	6.4%	5.7*10^6^	9.6%	7.6*10^6^	5.4%
Li-Hep (0, H, L)	n = 35	6.7*10^6^	18.5%	4.7*10^6^	19.4%	7.3*10^6^	14.1%
Serum (0, H, L)	n = 33	6.0*10^6^	8.5%	4.2*10^6^	10.2%	7.0*10^6^	6.4%

High variations in recovery of CAAPs in lithium-heparin plasma derived most likely from inconsistent responses of all three internal standards in lithium-heparin plasma.

### Stability

Stability of analytes in processed samples was determined at six independent days for a time span of 20 h in the autosampler. Deviations of standing samples to freshly measured samples were below 19% at the LLOQ and below 9% for other QC levels for each analyte, respectively (Additional file 4). Stability of analytes in biological matrix was determined by consecutive cycles of freezing and thawing as well as extended intervals at room temperature from thawing to processing (approximately one hour). Deviations of either freeze/ thaw treatment or extended benchtop time at room temperature compared to freshly processed QCs did not exceed ± 10% (CV) and mean accuracies were within 98–117, respectively (Additional file 4).

### Quantification of CAAPs in healthy humans and comparison between different whole blood-derived specimens

**Figure 1:**
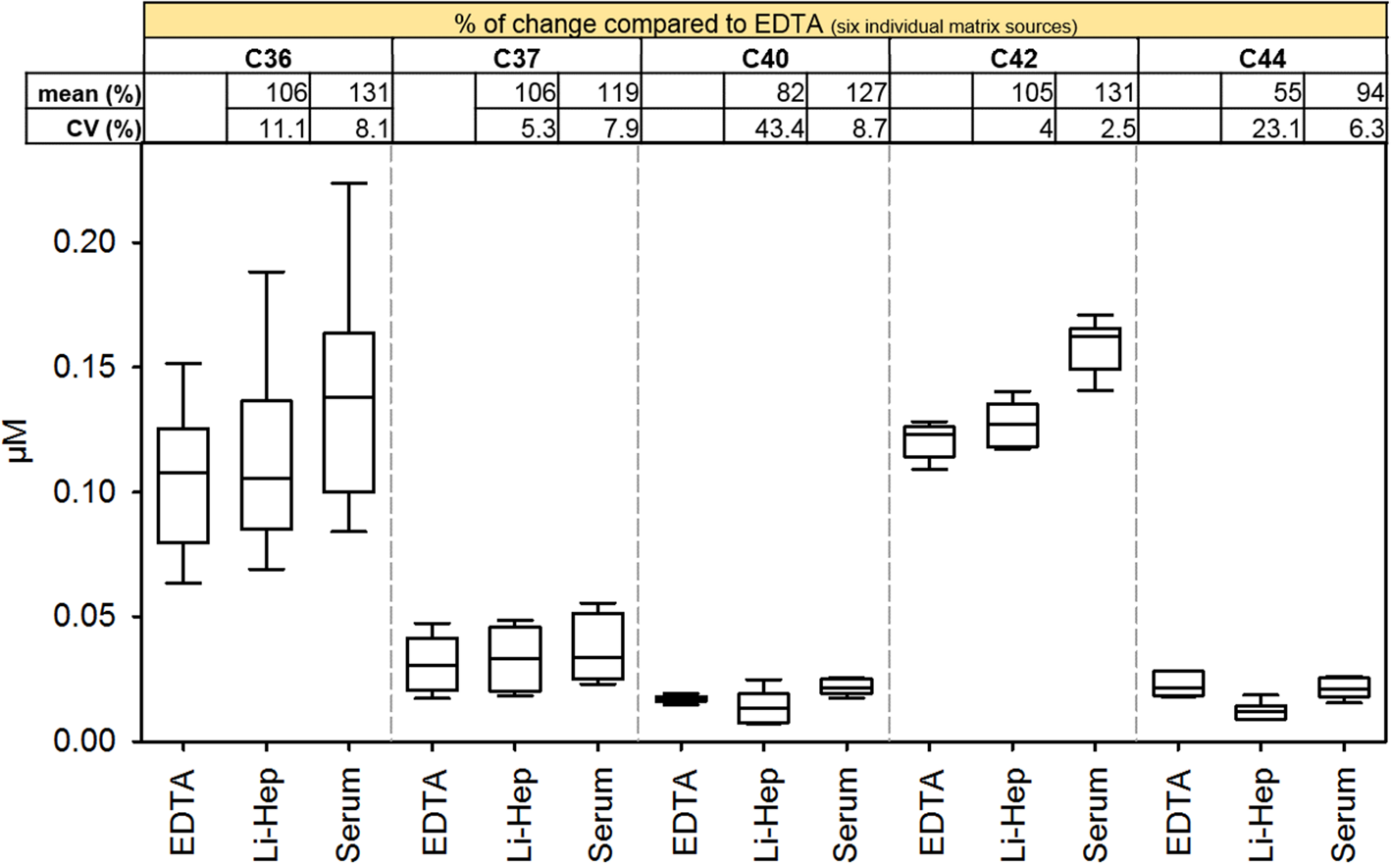
Comparison of CAAP levels in EDTA plasma, lithium-heparin plasma and serum. Relative changes (compared to EDTA plasma) were calculated for each donor individually (n = 6) before mean accuracy (%) and CV (%) were calculated.

Using the current MRM-based method we were able to quantify concentrations at baseline levels from healthy donors (n = 6) for five out of nine CAAPs investigated (namely C36, C37, C40, C42 and C44; Fig. 1).

Absolute concentrations of C36, C37, C40, C42 and C44 in EDTA plasma were 0.105 ± 0.030 µM, 0.031 ± 0.011 µM, 0.017 ± 0.001 µM, 0.121 ± 0.007 µM and 0.023 ± 0.005 µM, respectively (mean of n = 6 ± standard derivation). Since accuracy and precision of recovery rates in EDTA plasma exhibit best results among three tested blood-derived specimens, CAAP concentrations in lithium-heparin and serum were compared relative to EDTA plasma. Concentrations of CAAPs found in different matrices from same healthy donors revealed that the relative change of concentrations is consistent for serum compared to EDTA plasma (range of CV for five detected CAAPs: 2.5–8.7%; Fig. 1). However, mean serum concentrations differ between 94 and 131% compared to EDTA, depending on the respective CAAP. C36, C37 and C42 measured in lithium-heparin plasma also showed acceptable variation in relative concentrations compared to EDTA plasma (11.1%, 5.3% and 4% CV, respectively) and comparable mean concentrations compared to EDTA (between 105 and 106%). However, C40 and C44 showed unacceptable high variations among individuals (43.4 and 23.1%) and mean concentrations were much lower in lithium-heparin plasma (82 and 55%) compared to EDTA.

### Clinical applications

**Figure 2:**
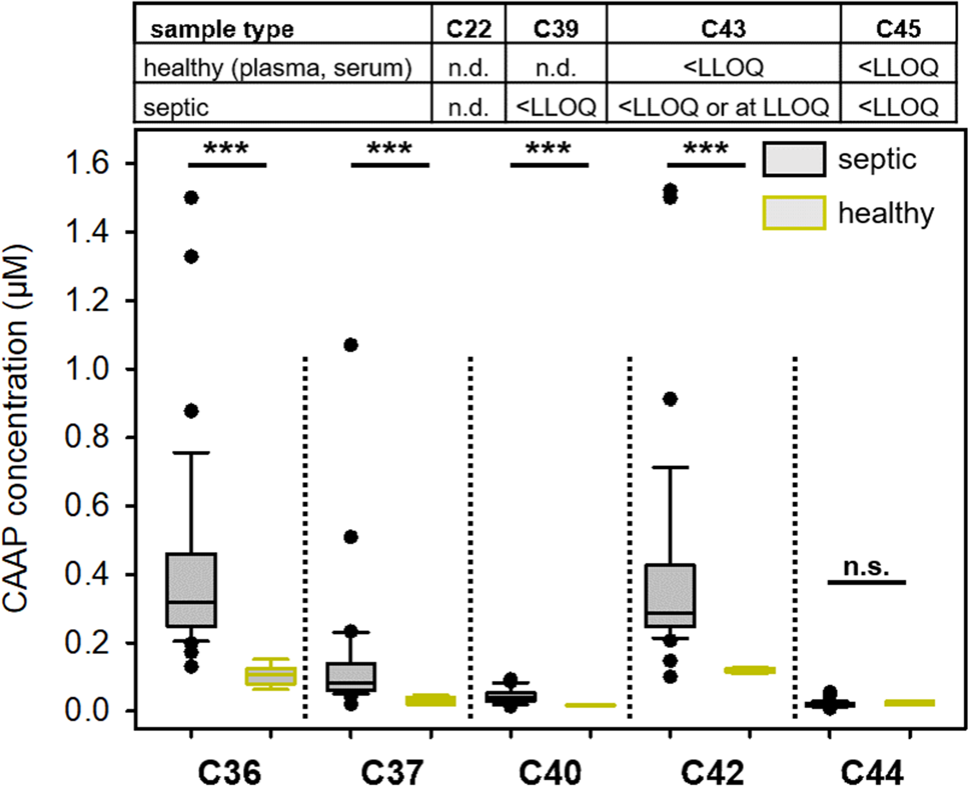
CAAPs abundance in healthy individuals (n = 6) and septic patients (n = 36, cohort A). Data are based on measurements of EDTA plasma samples, which were obtained from patients within the first three days after onset of sepsis. Statistical analysis was conducted using Mann–Whitney Rank Sum Test. *** *P* < 0.001, n.d. not detected, n.s. not significant.

Since CAAPs can be generated from endogenous enzymatic activity associated with inflammation as well as by pathogens frequently causing septic shock, a clinical patient’s cohort suffering from sepsis was chosen to initially investigate CAAP profiles during infectious-induced systemic inflammation. EDTA plasma was used because it proved to be the most suitable blood-derived specimens during method validation. Concentrations of C36, C37, C40 and C42 were significantly higher in the sepsis cohort compared to healthy control samples (*P* < 0.001, respectively, Fig. 2). C44 in septic patient samples remains on healthy concentration level (*P* = 0.229). C22, C39, C43 and C45 were absent or below LLOQ.

Based on median values for each CAAP concentration, sepsis patients exhibit between 2.3 and 3.0 times higher concentrations of C36, C37, C40 and C42 than healthy individuals. Among the significantly increased CAAPs in sepsis samples compared to healthy individuals (Fig. 2), we have investigated the validity of C36, C37, C40 and C42 as potential biomarker for sepsis using ROC analysis (Table 5). C36 and C42 resulted in AUROC value of above 97% and high sensitives (97%) and specificities (100%), respectively. Classifications of C37 and C40 were less sensitive and specific for discriminating sepsis from healthy patients.

**Table 5:** Accuracy of selected CAAPs for their potential to discriminate sepsis patients from healthy individuals. The data set used was the same as in Fig. 2.

CAAP	AUC [95% CI]	Cutoff^a^ (µM)	Sensitivity (n^+^/n)	Specificity (n^-^/n)
C36	99% [98–101%]	0.162	97% (35/36)	100% (6/6)
C37	97% [92–102%]	0.048	97% (34/36)	71% (5/6)
C40	96% [91–102%]	0.020	88% (35/36)	50% (1/6)
C42	97% [92–103%]	0.137	97% (35/36)	100% (6/6)

^a^ Cutoffs are derived from Kolmogorov–Smirnov statistics. AUC: area under the receiver operating characteristics curve; CI: confidence interval; n^+^: measurements with positive test results; n^-^: measurements with negative test results; n: all measured values.

**Figure 3:**
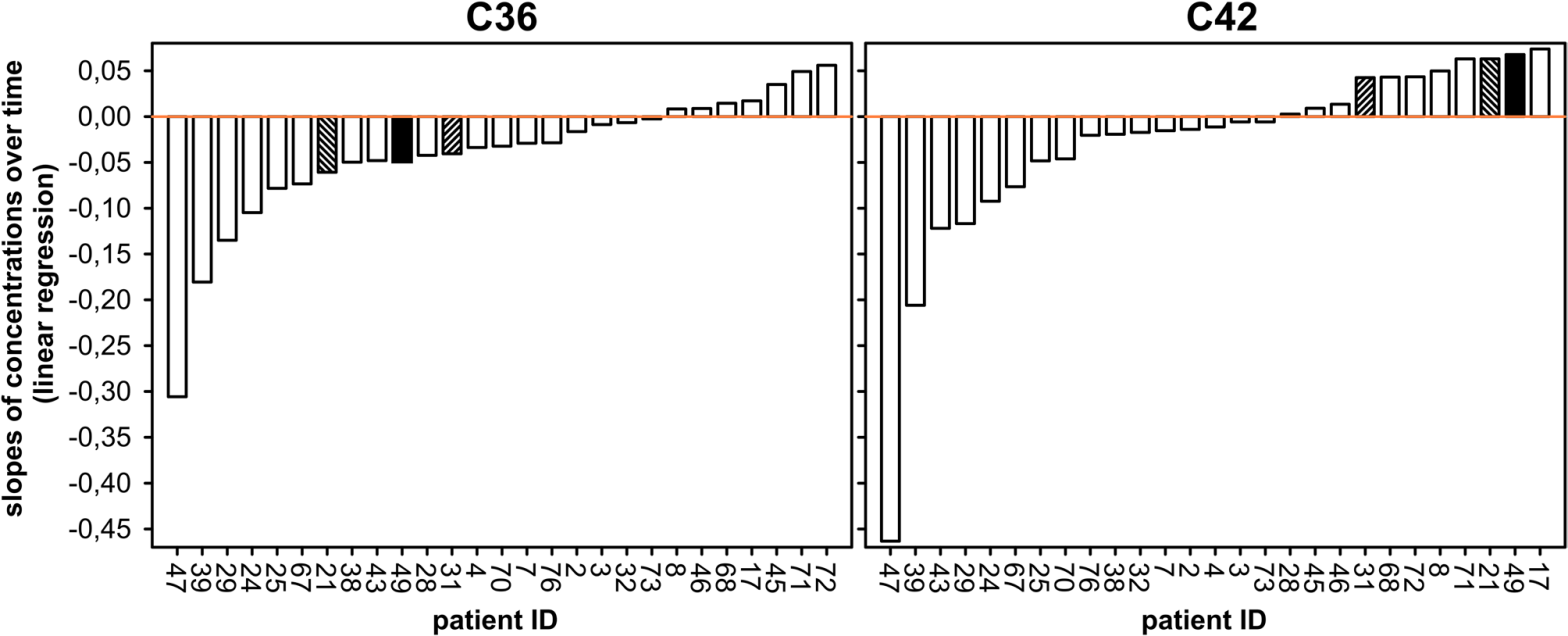
Development of C36 und C42 concentrations in septic patients. To obtain trends over time, linear regression analyses were conducted for patients with at least three samples collected during their stay at the ICU (cohort B, n = 27). Patients exhibiting opposite trends for C36 and C42 are highlighted.

For the two most abundant CAAPs C36 and C42, we have also analyzed their plasma concentrations in septic patients during the first week of treatment at the ICU (Fig. 3, n = 27).

Over the course of the disease, changes in concentrations of C36 and C42 vary greatly within the patient cohort; with C36 concentrations tending to decrease in the majority of cases (Fig. 3). We have further analyzed the development of C36 and C42 concentrations towards striking features among the sepsis cohort and we found that three out of 27 patients exhibit opposite trends for C36 and C42 concentrations over time (patient ID 21, 31 and 49; Fig. 3). For these three cases, C42 increases while C36 decreases over time.

## Discussion

Since the role of CAAPs as biomarker has been proposed for many diseases (^36,38,39,42,46^, see also Introduction), the proposed method is of important and broad clinical relevance. To this end, we also provide an overview about the current state of knowledge for CAAPs identified in vitro and in vivo (Table 6). In addition to CAAP generating conditions already described in the literature (Table 6), a vital role of CAAPs in other clinical contexts such as cancer progression^18,45,56,57^ and viral infections can be assumed due to increased MMP activities in the course of these diseases. For example, increased serum concentrations of neutrophil elastase (C36-generating enzyme) and MMP-12 (C42-generating enzyme) have been detected in severe COVID-19 patients^58^. Biomarkers that allow to quantify systemic inflammation can become an essential tool for diagnosis as well as for monitoring the therapy of systemic inflammatory disorders, but are rarely available yet^59^.

**Table 6:** Summary of known sources for CAAPs. ^a^in brackets cleavage site numbering according to 418 amino acid sequence length of A1AT including precursor molecule of 24 amino acids^60^ using one-letter-code for amino acids.

CAAP^a^	in vivo (human)	in vitro human tissue/ cells	in vitro assays using purified enzymes		References
			Enzyme name	Enzyme host^b^	
C45 (G^373^A)	Not detected in plasma or serum of healthy or septic individuals				Current study
			Glycyl endopeptidase	*Carica papaya*	^29,61^
C44 (A^374^M)	Plasma & serum (no difference between healthy and septic individuals)				Current study
		Placenta			^43,44^
		BALF (higher concentrations in premature infants with bronchopulmonary dysplasia)			^37^
			MMP-11	human endogenous	^6^
			Adamalysin II	*Crotalus adamanteus*	^29,62^
C43 (M^375^F)	Not detected in plasma or serum of healthy or septic individuals				Current study
			Thermolysin	*Bacillus thermoproteolyticus*	^61^
C42 (F^376^L)	Plasma & serum (higher concentrations in sepsis cohort than healthy individuals)				Current study
	EDTA plasma (higher concentrations in sepsis cohort than SIRS, HIV or neutropenia)				^46^
	NAF (only found in woman with breast cancer and among those only found in affected breast)				^45^
			MMP-1	Human endogenous	^7,8^
			MMP-7	Human endogenous	^9–11^
			MMP-8	Human endogenous	^12–14^
			MMP-9	Human endogenous	^12,14^
			MMP-12	Human endogenous	^15,16^
			MMP-13	Human endogenous	^63^
			MMP-25	Human endogenous	^17^
			MMP-26	Human endogenous	^18,19^
			Periodontain	***Porphyromonas gingivali***s	^26,27^
			Aureolysin	***Staphylococcus aureus***	^27,28^
			Thermolysin	*Bacillus thermoproteolyticus*	^27,28,61^
			Papain	*Carica papaya*	^29,61^
C40 (E^378^A)	Plasma & serum (higher concentrations in sepsis cohort than healthy individuals)				Current study
			Cathepsin L	Human endogenous	^20^
			Periodontain	***Porphyromonas gingivali***s	^26,27^
			Glutamyl endopeptidase	***Staphylococcus aureus***	^28^
			Aureolysin	***Staphylococcus aureus***	^27^
			Staphopain A	***Staphylococcus aureus***	^27–29^
			Pseudolysin	***Pseudonomas aeruginosa***	^27^
C39 (A^379^I)	Not detected in plasma or serum of healthy or septic individuals				Current study
			Der p1 allergen	*Dermatophagoides pteronyssinus*	^33^
C37 (P^381^M)	Plasma & serum (higher concentrations in sepsis cohort than healthy individuals)				Current study
			MMP-1	Human endogenous	^8^
			MMP-3	Human endogenous	^7,10,21^
			MMP-7	Human endogenous	^10,11^
			MMP-8	Human endogenous	^13,14^
			MMP-9	Human endogenous	^14^
			MMP-12 (mice)	Human endogenous	^16^
			MMP-13	Human endogenous	^63^
			MMP-25	Human endogenous	^17^
			MMP-26	Human endogenous	^18,19^
			Pseudolysin	***Pseudomonas aeruginosa***	^27,30^
C36 (M^382^S)	Plasma & serum (higher concentrations in sepsis cohort than healthy individuals)				Current study
	Spleen & bile (not found in plasma)				^40^
	BALF. Upregulated in patients with acute exacerbations idiopathic pulmonary fibrosis				^38^
	Gingival crevicular fluid (putative biomarker for GCF). Higher concentration than in healthy group				^39^
		Supernatants of activated (LPS-stimulated) neutrophils (not detected in unstimulated controls)			^64^
		Lung tissue (however, not correlated to COPD)			^41^
		Carotid artery tissue (symptomatic carotid artery stenosis undergoing carotid endarterectomy)			^42^
		BALF of premature infants			^37^
			Neutrophil Elastase	Human endogenous	^5^
			Cathepsin L	Human endogenous	^20^
			MMP-3	Human endogenous	^10^
			Prostate-specific antigen	Human endogenous	^22,23^
			Mesotrypsin (only Pittsburgh variant (M^382^ > R))	Human endogenous	^24^
			High temperature requirement A1	Human endogenous	^25^
			Serralysin	***Serratia marcescens***	^27,31^
			EspPα	enterohemorrhagic ***Escherichia coli***	^32^
			Seaprose	*Aspergillus melleus*	^34^
			Endopeptidase (ragweed)	*Ambrosia artemisiifolia*	^35^
			Papain	*Carica papaya*	^27,29,61^
			Trypsin	*Streptomyces erythraeus*	^65^
			bovines trypsin	bovine	^65^
			porcine pancreatic elastase	porcine	^66^
			α-chymotrypsin	bovine	^61^
C22 (F^396^L)	Not detected in plasma or serum of healthy or septic individuals				Current study
	Urine (higher concentration in glomerular kidney disease patients compared to healthy)				^36^
		BALF (higher concentrations in premature infants with bronchopulmonary dysplasia)			^37^
			MMP-7	human endogenous	^11^

^b^bold: human infectious microbes, underlined: not human infectious organism. A1AT: α1-Antitrypsin; CAAP: C-terminal region of α1-Antitrypsin; MMP: matrix metalloproteinase; BALF: bronchoalveolar lavage fluid. NAF: nipple aspiration fluids.

Due to the role of C42 as biomarker in systemic infection^46^ and thus its potential application as sepsis biomarker in routine diagnostics, method development had focused on a rapid workflow. In contrast to the original LC–MS/MS method only targeting C42^WT^ and C42^SNP^^46^, the current method possesses a rapid workflow and optimized chromatographic conditions for eighteen CAAP analytes. The entire process from sample preparation to quantitative results can be completed in less than one hour (30 min. sample preparation, 12 min. LC run). Working ranges were optimized based on results for carryover and specificity as well as concentrations detected in healthy and septic individuals. Concentration-dependent responses of all investigated CAAPs were linear across more than two orders of magnitude. Therefore, time-consuming steps for sample preparation (*e.g.* extraction or dilution), which are usually applied, are not necessary. In addition to the more efficient workflow, quantification of CAAPs in healthy individuals can now also be achieved (Fig. 1) due to increased sensitivity of the new method compared to the previous method^46^. Finally, tests determining accuracy and precision (within-run repeatability and between-runs) of back-calculated CAAP concentrations using QC samples were successfully passed (Table 2).

Even though results of specificity between WT and SNP of some CAAPs are higher than 20% of the respective LLOQ (Additional file 2), we assume that the chance of incorrectly determined concentrations due to interferences from other CAAPs is negligible due to several reasons. First, concentrations below the LLOQ are generally not taken for final data analysis. Second, WT- and SNP-variant would both considered in one sample in case of heterozygote genotype of rs1303 polymorphism which is true for only 37% of European American individuals^67^). Third, among the analytes which exceed limits of specificity measures according to guidelines (maximum 20% of the LLOQ)^54,55^, C36 is only one for which we have actually measured concentrations near the ULOQ. Fourth, back-calculated concentrations of WT and SNP which differ greater than 30% from each other are not considered as heterozygote genotype.

Combining results from recovery rates and absolute concentrations in healthy donors (Table 3, Fig. 1), EDTA plasma and serum are appropriate matrices to quantitatively measure CAAPs, whereas lithium-heparin plasma revealed results with high variations among individuals. Using lithium-heparin plasma for CAAP measurements is hence not suggested. Irrespective of the matrix, comparison of respective CAAP concentrations among different clinical entities (*e.g.* healthy versus diseased) should be performed within the same matrix, if possible. Nevertheless, for data analysis it is recommended to define reference ranges for each CAAP concentration in each matrix.

The presence of C36, C37, C40, C42 and C44 in healthy individuals (Fig. 2) is in agreement with knowledge from the literature as those CAAPs have been reported to be generated endogenously in humans (Table 6). However, apart from our own previous study^46^, this is to the best of our knowledge the first report about quantitatively measured C-terminal cleavage products of alpha-1-antitrypsin in blood-derived samples. Johansson and colleagues analyzed CAAPs in plasma, bile and spleen using liquid–liquid extraction and amino acid sequencing techniques^40^. However, they have detected C36 in bile and spleen only but not in plasma. Four peptides (C22, C39, C43, C45) were not detected or below the LLOQ (Fig. 2). Their absence in plasma is partly in agreement with knowledge from the literature (Table 6). C39, C43, and C45 have indeed never been detected in vivo; and in vitro identifications of C43^61^ and C45^29,61^ have been correlated only to enzymes that are not associated with human diseases yet. C39, however, has only been found in vitro as potential cleavage product associated with allergic reactions^33^ whereas C22 has been identified in vivo in urine^36^ and BALF^37^. In addition to the higher concentrations of C42^46^ in septic patients compared to healthy individuals (Fig. 2) our results demonstrate that C36, C37 and C40 seem to play a crucial role in infectious-induced systemic inflammation as well and thus might also have the potential to serve as sepsis biomarkers in blood-derived samples. And indeed, the validity of C36 and C42 as sepsis biomarker was confirmed by their high sensitivities and specificities, respectively (Table 5). C36 experiences the greatest change among CAAPs measured in septic patients compared to concentrations measured in healthy individuals (n = 36, Fig. 2). This observation might be related to MMP activity since it has been shown that higher plasma levels of MMP-3 are associated with septic shock and mortality^68^. C22, C39, C43 and C45 seem to play only a minor, if any, role during systemic inflammation because they were not detected or below the LLOQ in septic patients (Fig. 2). C36 and C42 concentrations of samples from septic patients (n = 27) change over time (Fig. 3) suggesting that enzymatic activities and/or elimination kinetics are related to the course of infectious-induced systemic inflammation. It is in agreement with knowledge from the literature that formations of C36 and C42 are mediated through distinct enzymatic activities, respectively (Table 6). Of note, for three patients (ID 21, 31 and 49) we detected clearly opposite trends for C36 (decrease) and C42 (increase) concentrations over time (Fig. 3). Decrease of C36 is followed by high initial C36 concentrations; moreover all three cases were affected by pneumonia (site of sepsis infection) with very high SOFA scores at admission (patient 21: 11; patient 31: 15; patient 49: 15). It is well known that increased activity of neutrophil elastase is involved in the pathogenesis of various lung diseases such as pneumonia (see reviews for example^69,70^). C36 can be formed by neutrophil elastase^5^ and has already been found in vivo in lung-correlated tissues^37,38,41^. Further investigation and studies are needed to uncover potential correlations of CAAPs with, for example, site of infection, organ failure or specific enzymatic activities.

## Conclusion

Since the generation of CAAPs is multifaceted and they have been found in a variety of biomaterials and in response to a wide range of pathological changes, our method provides a useful clinical tool for many diseases. We are confident that the presented method can be easily adopted towards a variety of biological materials in order to specifically study CAAP levels at sites of generation such as placenta, BALF, bile or urine as well.

## Material and methods

### Selection of target compounds

**Figure 4:**
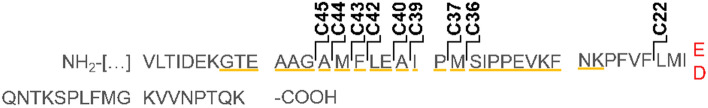
Cleavage sites of selected CAAPs within the C-terminal amino acid sequence of A1AT. CAAPs are named after their respective amino acid lengths after cleavage. Yellow: 25 residue reactive centre loop of A1AT (UniProtKB). Red: single nucleotide polymorphism rs1303^53^ in the CAAP sequence (E > D^376^).

The scientific literature search was conducted manually for known cleavage sites of A1AT. Among the variety of C-terminal peptides of A1AT (CAAPs) a panel of nine full-length CAAPs was selected (Fig. 4) according to their experimental evidence. Fragments that were only predicted as well as intermediate fragments in the C-terminal region of A1AT (such as VIRIP) were not collected.

Due to a minor allele frequency for the single nucleotide polymorphisms (SNP) of A1AT (rs1303, 28%) within the investigated C-terminal amino acid sequence, the final CAAP panel consists of eighteen analytes (wild type [WT] and SNP for each CAAP length). Authentic CAAPs and internal standards were obtained from sb-PEPTIDE (SmartBioscience SAS, Saint Egrève, FRANCE) as lyophilized peptides in aliquots. C22, C37 and C42 containing isotopic labelled lysine (^13^C^15^N) were used as internal standards (IS) for quantification (C22^IS^, C37^IS^, C42^IS^).

### Sample preparation

Lyophilized authentic peptide standards were dissolved and diluted in a 70 g/L Albumin (from human serum, lyophilized, ≥ 99%, Sigma-Aldrich) in PBS (Sigma-Aldrich, St. Louis, MO, USA) solution. Internal standards C37^IS^ and C42^IS^ were dissolved in Milli-Q water (Millipore, Brussels, Belgium) containing 0.1% (*v/v*) formic acid (≥ 95%, Sigma-Aldrich) whereas C22^IS^ was only dissolved in ultrapure water. Mixtures of CAAPs used as calibration standards (calibrants) and quality control samples (QCs) were prepared as stock solution to a final concentration of 14 µM, respectively, (expect C40^SNP^ which was 5 µM) with subsequent serial dilutions. Calibrants and QCs were obtained from separate serial dilutions. Internal standard solution was prepared by adding equal volumes of each IS yielding a final concentration of 0.8 µM, respectively. All samples used for method development and validation were prepared and stored in 0.5 mL Protein LoBind Tubes (Eppendorf AG, Hamburg, Germany) in aliquots at − 80 °C until use.

For LC–MS/MS analyses, 10 µL of internal standard solution was added to 35 µL of calibrant, QC or study sample and gently mixed by pipetting. This mixture was then briefly centrifuged (10 s; 16,000* g*; + 10 °C; model 5415R, Eppendorf AG) before 90 µL of cooled methanol (≥ 99.9%, Carl Roth, Karlsruhe, Germany) was added and briefly vortexed subsequently. After centrifugation (10 min; 16,000* g*; 10 °C) 100 µL of supernatant was transferred into 2 mL brown glass autosampler vials using 200 µL glass inserts (Wicom, Heppenheim, Germany).

### LC–MS/MS analyses

For chromatographic separation, a Shimadzu HPLC system (Duisburg, Germany) was used which is equipped with a binary pump (LC20AB), a thermostatic autosampler (SIL20AC) and a thermostatic column compartment (CTO20AC). 5 µL sample was injected from the autosampler (maintained at 10 °C) and loaded on the UHPLC column (bioZen 3.6 µM Intact C4, 2.1 ˣ 100 mm, Phenomenex, Aschaffenburg, Germany). The column oven was maintained at 45 °C; solvent A was 0.1% formic acid in ultrapure water (*v/v*) and solvent B was 0.1% formic acid in LC–MS Grade acetonitrile (*v/v*, ≥ 99.95%, Carl Roth). A step gradient was applied as follows: 5–25% B from 0–1 min., 25–46% B from 1–6 min., 46–85%B from 6–6.5 min., 85% B from 6.5–8.5 min., 85–5% B from 8.5–9.0 min. and 5% B from 9–12 min. The flow rate was 350 µL/min from start to 6.5 min. and 10.6 to 12 min., respectively, and 600 µL/min from 7.0 to 10.5 min.

For mass spectrometry detection, a Triple Quad 5500 + (AB SCIEX, Framingham, MA) in multiple reaction monitoring (MRM) scan type was used with Q1 at low and Q3 at unit resolution. The instrument was equipped with a Turbo V™ ion source and operated in positive electrospray ionization mode with source parameters as follows: curtain gas = 35, collision gas = 8, ionSpray voltage = 5500, temperature = 700 °C, nebulization gas (GS1) = 70, drying gas (GS2) = 70.

Two transitions were monitored for each compound (quantifier, qualifier) and one for the internal standards using an intensity threshold of 50 cps. Corresponding compound-dependent parameters were optimized for each transition (Table 7) whereas entrance potential was 10 V for all transitions. Dwell time was 35 ms for all quantifier and 10 ms for all qualifier. A representative chromatogram is shown in Additional file 1.

**Table 7:** Compound-dependent parameters for the detection and quantification of CAAPs.

Compound (mass^a^)		RT-window [min]	Precursor		Product [*m/z*]	DP [V]	CE [V]	CXP [V]	IS
			*m/z*	charge					
C22^WT^ (2502.33)	Quantifier	3.3–3.5	626.8	[M + 4H]^4+^	754.1	110	25	7	C22^IS^
	Qualifier		626.7		217.3	125	30	20	
C22^SNP^ (2488.32)	Quantifier	3.3–3.5	623.3		749.4	110	25	7	
	Qualifier		623.3		217.3	125	30	20	
C36^WT^ (4132.23)	Quantifier	4.2–4.7	689.84	[M + 6H]^6+^	787.8	105	20	6	C37^IS^
	Qualifier		689.84		173.4	125	50	15	
C36^SNP^ (4118.21)	Quantifier	4.1–4.6	687.49		785	105	20	6	
	Qualifier		687.49		173.4	125	20	15	
C37^WT^ (4263.27)	Quantifier	4.3–4.8	711.74	[M + 6H]^6+^	787.8	105	30	6	
	Qualifier		854.09	[M + 5H]^5+^	984.8	125	40	20	
C37^SNP^ (4249.25)	Quantifier	4.3–4.8	709.5	[M + 6H]^6+^	785.1	105	30	6	
	Qualifier		851.26	[M + 5H]^5+^	981.1	125	40	20	
C39^WT^ (4473.40)	Quantifier	4.4–5.0	747.06	[M + 6H]^6+^	728.2	105	30	6	
	Qualifier		896.05	[M + 5H]^5+^	984.5	125	40	15	
C39^SNP^ (4459.39)	Quantifier	4.3–4.9	744.63	[M + 6H]^6+^	785.1	105	35	6	
	Qualifier		744.63		129.2	125	40	15	
C40^WT^ (4544.44)	Quantifier	4.4–5.0	758.25	[M + 6H]^6+^	728.2	105	20	6	C42^IS^
	Qualifier		910.38	[M + 5H]^5+^	873.6	125	30	20	
C40^SNP^ (4530.42)	Quantifier	4.3–4.9	756.45	[M + 6H]^6+^	725.9	105	20	6	
	Qualifier		907.51	[M + 5H]^5+^	870.8	125	30	10	
C42^WT^ (4786.57)	Quantifier	4.6–5.2	799.2	[M + 6H]^6+^	873.6	105	25	6	
	Qualifier		958.6	[M + 5H]^5+^	1091.9	125	40	20	
C42^SNP^ (4772.55)	Quantifier	4.5–5.1	796.75	[M + 6H]^6+^	870.7	105	25	6	
	Qualifier		956	[M + 5H]^5+^	1088.4	125	40	15	
C43^WT^ (4933.64)	Quantifier	4.9–5.5	823.65	[M + 6H]^6+^	873.4	105	25	6	
	Qualifier		988.28	[M + 5H]^5+^	1091.9	125	40	15	
C43^SNP^ (4919.62)	Quantifier	4.8–5.4	821.3	[M + 6H]^6+^	870.8	105	25	6	
	Qualifier		985.4	[M + 5H]^5+^	1088.2	120	40	15	
C44^WT^ (5064.68)	Quantifier	5.0–5.6	845.35	[M + 6H]^6+^	873.9	105	35	6	
	Qualifier		1014.5	[M + 5H]^5+^	1091.9	125	40	15	
C44^SNP^ (5050.66)	Quantifier	5.0–5.5	843.08	[M + 6H]^6+^	870.7	100	35	6	
	Qualifier		1011.59	[M + 5H]^5+^	1088.1	125	40	15	
C45^WT^ (5135.71)	Quantifier	5.1–5.7	857.59	[M + 6H]^6+^	873.3	100	35	6	
	Qualifier		1028.58	[M + 5H]^5+^	1091.9	125	40	15	
C45^SNP^ (5121.70)	Quantifier	5.1–5.6	855.19	[M + 6H]^6+^	871.0	100	35	6	
	Qualifier		1025.85	[M + 5H]^5+^	1088.1	120	40	15	
C22^IS^ (2516.37)	Quantifier	3.3–3.5	630.06	[M + 4H]^4+^	756.4	110	25	7	-
	Qualifier		630.06	[M + 4H]^4+^	224.4	125	30	20	
C37^IS^ (4277.30)	Quantifier	4.3–4.8	714.01	[M + 6H]^6+^	790.6	105	30	6	
	Qualifier		856.76	[M + 5H]^5+^	988.0	125	40	15	
C42^IS^ (4807.62)	Quantifier	4.7–5.2	802.72	[M + 6H]^6+^	876.3	105	25	6	
	Qualifier			[M + 5H]^5+^	1095.1	125	40	15	

^a^ Theoretical monoisotopic mass (g/mol). DP = declustering potential, RT = retention time, CE = collision energy, CXP = collision cell exit potential, SNP = single nucleotide polymorphism, IS = internal standard.

### Bioanalytical method validation

*Working range of calibrants* was defined based on CAAPs detected in samples from healthy donors (set to approximate lower limit of quantification) and in samples from septic patients (set to middle to high calibration range) which were re-analyzed from a previously work^46^. Nine calibrants were used in total ranging from 0.01 µM to 1.5 µM thereby covering more than two orders of magnitude in concentration*. Linearity* was assessed determining mean linear regression coefficients of each calibration curve from 0.01 to 1.5 µM in eight independent analytical runs. However, respective LLOQs (lower limit of quantification) and ULOQs (upper limit of quantification) were defined individually based on natural occurrence (see above) and results from validation parameter directly affected individual working ranges (carry-over, specificity). Four QC samples were prepared and measured in total (QC1, QC2, QC3, and QC4) to cover the entire range of calibration. However, three QCs for each of the respective analytes were used for method validation. *Carry-over* was determined measuring pure albumin solutions prepared without internal standards (but equal amount and concentration of water/formic acid solution as regular internal standard solution, “blank albumin sample”) directly after the respective highest calibrants. Peak areas were compared to peak areas from respective LLOQ samples (prepared with regular internal standard solution). *Specificity *was assessed using single compounds at respective highest calibrant level which were prepared and calculated as described for carry-over. The specificity of analytes towards internal standards was determined using pure albumin solution with internal standard (“zero albumin sample”). *Accuracy and precision* was determined by back-calculated concentrations from repeated measurements of the same QC samples during one analytical run (*within-run repeatability*, n = 5) and from freshly prepared samples during five independent analytical runs (*between-run*, n = 5). Due to the unavailability of analyte-free biological matrix*, selectivity* of analytes were assessed in pure albumin solution containing internal standards. *Matrix effects* were determined calculating recovery rates and precision of each compound in human blood samples. Direct comparison between different anticoagulants (EDTA, lithium-heparin) and serum was performed using whole blood from the same human donor. Whole blood was collected in EDTA K_3_, Lithium-Heparin Plasma-Gel and Serum Gel Z S-Monovettes (Sarstedt, Nümbrecht, Germany) from six healthy donors (3f./ 3 m; age from 27 to 45 years), respectively, and prepared using standard centrifugal conditions (10 min., 2762 g, room temperature; model 5804R, Eppendorf AG) before storage at -80 °C. To minimize pre-analytical interferences, HIL indices (hemolysis, icterus, lipemia) were measured in lithium-heparin plasma as well as serum (Architect ci8200, Abbott Laboratories, Abbott Park, IL, USA) and results from both measures were valued as within normal ranges (data not shown). To determine *recovery rates in different blood matrices* spiked plasma was obtained by adding 5 µL of the spiking solution (peptide-mix standard in albumin: high [7 µM] and low [1 µM]) to 30 µL of different biological matrices from six healthy donors (EDTA plasma, lithium-heparin plasma and serum), respectively. Control samples (“0”) were pure albumin solutions added to biological matrices. Measurements were performed in duplicates. To determine recovery rates, accuracy was calculated by peak area ratios of analyte to internal standard using following equation: (area ratio of spiked sample) – (area ratio of endogenous concentration))/ (area ratio of spiked albumin sample). To evaluate the consistence of matrix effect among individual donors, precision of peak area ratios of analyte to internal standard was investigated for six individual donors in each of the biological matrix. To verify the *linear response* of CAAPs in different matrices, pool matrices consisting of equal volumes from each of the six donors was prepared and spiked with standard peptide mix (14 µM stock) before serial dilutions in the same pool matrix were conducted. Investigations of analyte *stabilities *were carried out using QC samples. Autosampler stability was determined by injecting each QC at the beginning of the run and 20 h later during six independent runs. The CV (%) of the back-calculated concentration (n = 2) was used to ensure autosampler stability. To determine stability of analytes after two cycles of freezing and thawing (“freeze/ thaw”) as well as after chilling for 60 min. at room temperature (“benchtop”) three QCs at two concentrations levels were used (QC2 and QC3), respectively. Samples were analyzed against freshly prepared QCs.

### Study cohort

To initially determine CAAP profiles in sepsis patients, a well-characterized set of study samples provided by the Department of Anesthesiology and Intensive Care Therapy (Jena University Hospital) was chosen^71^. All Patients were treated at the Intensive Care Unit (ICU) and blood samples were taken within 24 h after septic symptoms emerged and within the following seven days. For data analysis and interpretation of CAAPs profiles, two subgroups were selected (cohort A and cohort B). Above all, we were interested in CAAP profiles to investigate their potential as early biomarkers. Therefore, we grouped all patients of whom blood samples were available within three days after sepsis onset (cohort A). Next, we were interested in CAAPs development. To this end, we grouped all patients of whom blood samples were available at least three different days after sepsis onset and during their stay at the ICU (cohort B). Patient characteristics of the subgroups are listed in Table 8.

**Table 8:** Patient characteristics. Severe sepsis/ septic shock was diagnosed according to the ACCP/SCCM criteria^72^ and corresponds to the Sepsis-3 definition^73^.

	Cohort A, *n* = 36	Cohort B, *n* = 27
Age, median (range)	66 (31–84)	66 (31–84)
Female, *n* (%)	14 (39)	8 (30)
28-day-survival, *n* (%)	27 (75)	22 (81)
SOFA at day of study admission^a^, median (25–75 percentile range)	10 (7–13)	10 (9–13)
**Site of infection, *n* (%)**		
Abdominal	16 (44.4)	9 (33.3)
Pneumonia	10 (27.8)	11 (40.7)
Soft tissue	–	2 (7.4)
Primary bacteremia	5 (13.9)	1 (3.7)
Endocarditis	3 (8.3)	3 (11.1)
Urogenital	2 (5.6)	1 (3.7)
**Microbial-positive infection**, *n* (%)	**24** (67)	**16** (59)
Positive blood culture	9 (37.5)	5 (31)
Multiple microbial-positive	5 (20.8)	5 (31)
*S. aureus*	4 (16.7)	3 (18.8)
*E. coli*	5 (20.8)	4 (25)
*P. aeruginosa*	3 (12.5)	–
*Enterococci*	–	–
others	5 (20.8)	3 (18.8)

Cohort A includes all patients of whom blood samples were available within three days after sepsis onset. Cohort B includes patients of whom blood samples were available at least three different days after sepsis onset and during their stay at the ICU.

^a^ When SOFA was not available at the day of study admission, SOFA was taken from the next day (n = 4).

### Data processing and statistical analysis

Analyst Software (version 1.6.2 and 1.7.1) was used for mass spectrometer data acquisition and processing. The raw data were imported into the quantification wizard tool, peak integration was reviewed individually and results were generated in relation to the respective internal standard. A 1/x*x weighted quadratic regression was used to calculate the concentrations of each of the eighteen compounds.

To calculate final concentrations for each of the nine CAAP lengths, concentrations of C42^WT^ and C42^SNP^ were used as reference compounds for determining individual phenotypes of rs1303 polymorphisms (homozygote WT, homozygote SNP or heterozygote). A tolerance of maximum 30% coefficient of variance was accepted between concentrations of C42^WT^ and C42^SNP^ for the heterozygote type.

Graphs and statistical analysis were performed using SigmaPlot 14.0. Measurements are presented as boxplots (median, 10/90th percentile) with error bars. For the classification of CAAPs as biomarkers, receiver operating characteristics analyses and cross tables were performed using SPSS statistics version 27 (IBM, New Armonk, NY).

### Ethics approval and consent to participate

The study was approved by the Ethics Committee of Jena University Hospital (protocol numbers: 2018–1145-Material, 2018-1145_1-Material, 4619–11/15) and conducted in accordance with the Declaration of Helsinki. All participants (or their legal guardians) gave their written informed consent for analyses and data collection on a consent documentation form.

## Supplementary Information

Supplementary Information.

## Data Availability

The datasets used and/or analysed during the current study are available from the corresponding author on reasonable request.
