# Supplementary material for: Multiplex quantification of C-terminal alpha-1-antitrypsin peptides provides a novel approach for characterizing systemic inflammation
Source: Sci Rep. 2022 Mar 9;12:3844. doi: 10.1038/s41598-022-07752-w (PMC8907207; doi:10.1038/s41598-022-07752-w)
Supplement: Supplementary file 1 — Supplementary Information. [file 41598_2022_7752_MOESM1_ESM.docx]

**Supplementary Figure S1**: Representative chromatogram of calibrant standards (top) and internal standards (bottom).


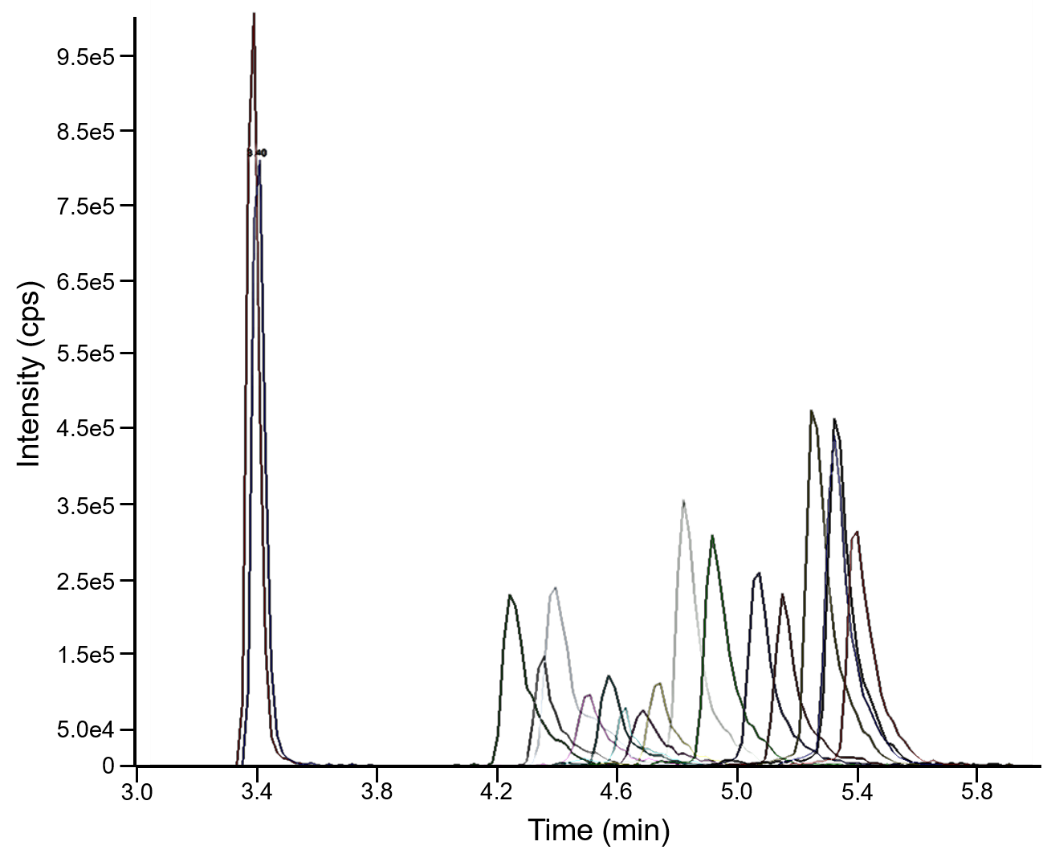


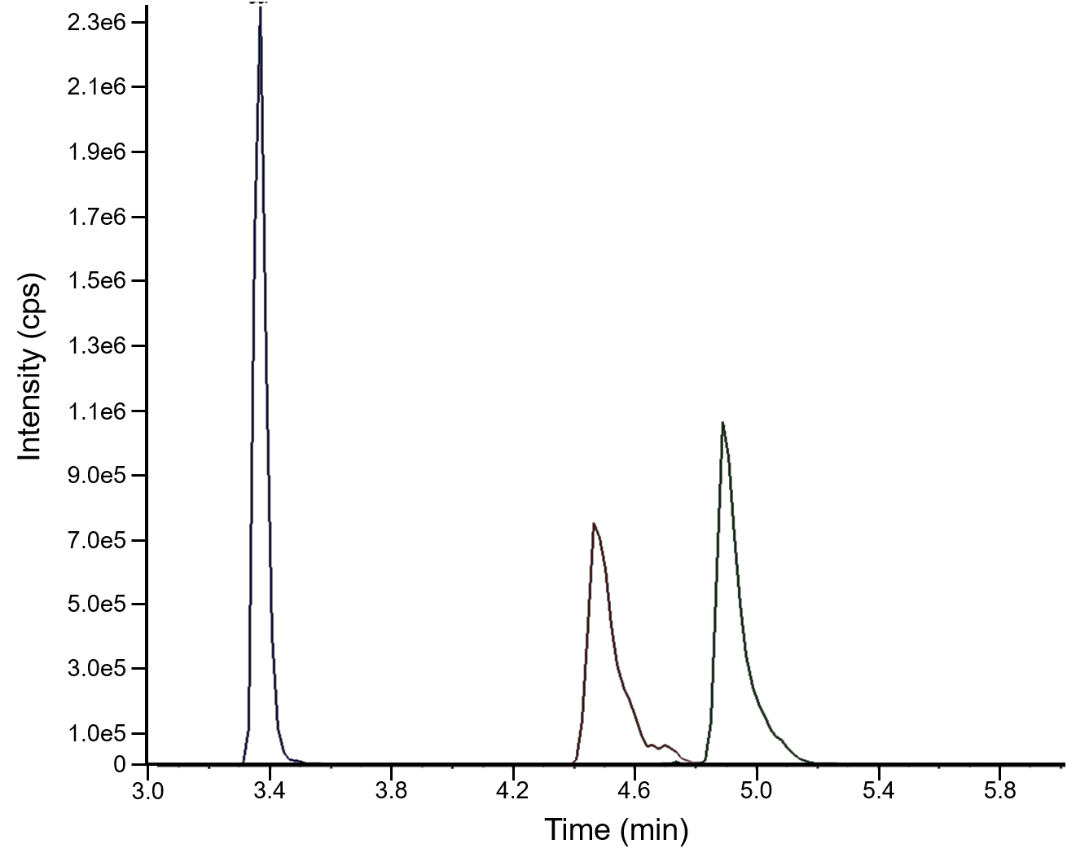


**Supplementary Figure S2**: Linear regression analyses of CAAPs in different blood-derived specimens and blank albumin matrix after serial dilution. Each CAAP is shown in wildtype (WT) and single nucleotide polymorphism variant (SNP).


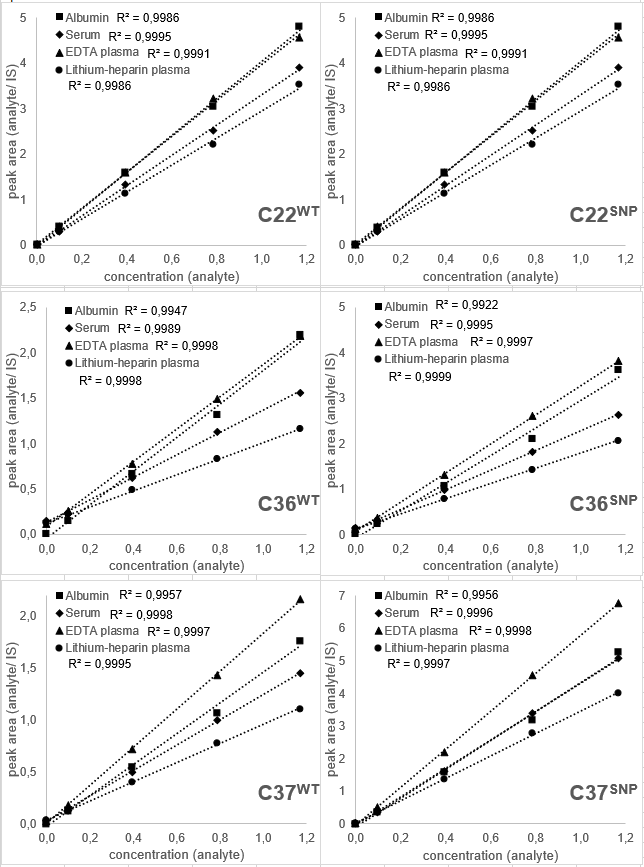


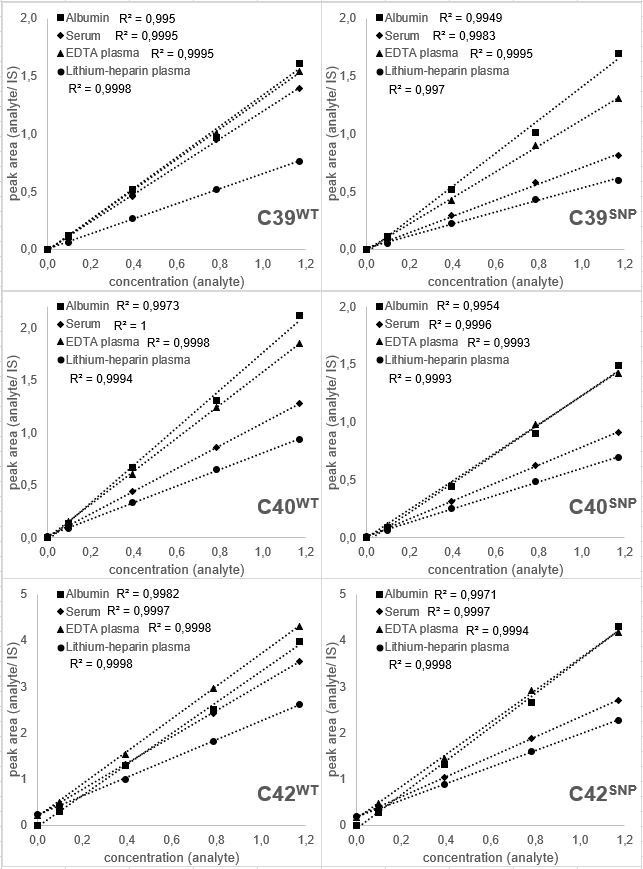


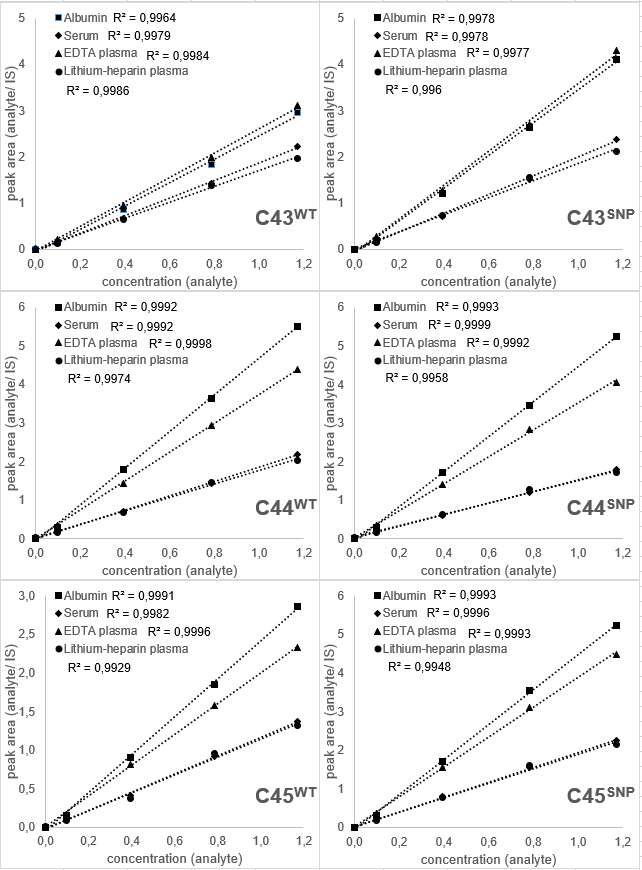


**Supplementary Table S1**: Specificity.


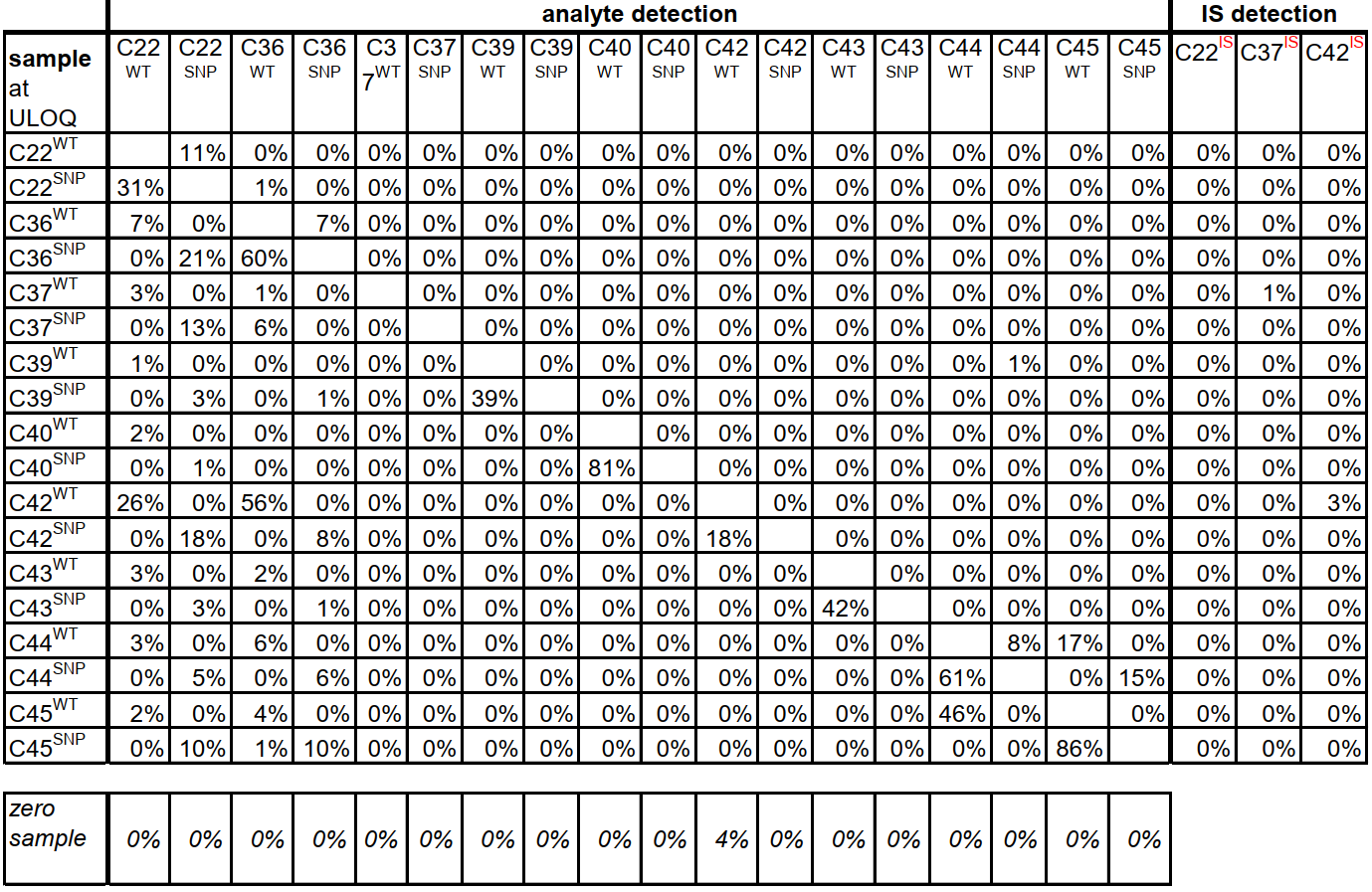


**Supplementary Table S2**: Stability of QCs.**a** autosampler stability. **b** freeze/thaw stability. **c** benchtop stability

| **a** |  | CV (%) within 20 hours autosampler time (11°C) | | | | | |
| --- | --- | --- | --- | --- | --- | --- | --- |
|  |  | **day1** | **day2** | **day3** | **day4** | **day5** | **day6** |
| C22^WT^ | QC1 | 3 | 2 | 2 | 2 | 5 | 4 |
|  | QC2 | 1 | 3 | 1 | 6 | 6 | 4 |
|  | QC3 | 2 | 3 | 3 | 7 | 3 | 1 |
| C22^SNP^ | QC1 | 0 | 8 | 2 | 1 | 3 | 6 |
|  | QC2 | 2 | 2 | 0 | 1 | 1 | 3 |
|  | QC3 | 1 | 1 | 1 | 1 | 2 | 3 |
| C36^WT^ | QC2 | 4 | 2 | 4 | 2 | 0 | 3 |
|  | QC3 | 1 | 2 | 0 | 6 | 1 | 2 |
|  | QC4 | 2 | 0 | 3 | 1 | 2 | 0 |
| C36^SNP^ | QC2 | 0 | 4 | 0 | 4 | 2 | 1 |
|  | QC3 | 4 | 1 | 2 | 5 | 2 | 0 |
|  | QC4 | 4 | 1 | 2 | 2 | 1 | 0 |
| C37^WT^ | QC1 | 4 | 3 | 1 | 8 | 4 | 5 |
|  | QC2 | 1 | 2 | 5 | 0 | 2 | 5 |
|  | QC3 | 1 | 3 | 4 | 2 | 0 | 2 |
|  | QC4 | 0 | 4 | 3 | 0 | 2 | 0 |
| C37^SNP^ | QC1 | 13 | 3 | 0 | 5 | 2 | 3 |
|  | QC2 | 4 | 0 | 4 | 1 | 2 | 7 |
|  | QC3 | 3 | 1 | 0 | 3 | 1 | 0 |
|  | QC4 | 0 | 2 | 0 | 0 | 0 | 1 |
| C39^WT^ | QC1 | 18 | 0 | 4 | 0 | 1 | 2 |
|  | QC2 | 1 | 8 | 2 | 2 | 0 | 1 |
|  | QC3 | 1 | 2 | 1 | 6 | 3 | 4 |
| C39^SNP^ | QC1 | 6 | 2 | 1 | 7 | 5 | 4 |
|  | QC2 | 0 | 1 | 2 | 2 | 0 | 0 |
|  | QC3 | 1 | 2 | 2 | 0 | 0 | 2 |
| C40^WT^ | QC1 | 4 | 7 | 1 | 3 | 4 | 5 |
|  | QC2 | 1 | 1 | 4 | 2 | 4 | 1 |
|  | QC3 | 0 | 4 | 4 | 0 | 0 | 3 |
| C40^SNP^ | QC1 | 3 | 4 | 6 | 3 | 4 | 2 |
|  | QC2 | 7 | 8 | 2 | 4 | 2 | 1 |
|  | QC3 | 2 | 3 | 1 | 1 | 0 | 1 |
| C42^WT^ | QC2 | 2 | 0 | 0 | 4 | 4 | 2 |
|  | QC3 | 0 | 1 | 2 | 1 | 1 | 1 |
|  | QC4 | 1 | 2 | 1 | 1 | 0 | 2 |
| C42^SNP^ | QC2 | 4 | 6 | 2 | 1 | 3 | 3 |
|  | QC3 | 1 | 2 | 0 | 2 | 3 | 1 |
|  | QC4 | 1 | 1 | 1 | 1 | 1 | 1 |
| C43^WT^ | QC1 | 0 | 5 | 5 | 2 | 2 | 2 |
|  | QC2 | 4 | 2 | 0 | 4 | 2 | 1 |
|  | QC3 | 2 | 1 | 0 | 2 | 3 | 3 |
| C43^SNP^ | QC1 | 2 | 2 | 1 | 1 | 3 | 2 |
|  | QC2 | 3 | 2 | 1 | 0 | 4 | 2 |
|  | QC3 | 1 | 2 | 2 | 2 | 1 | 1 |
| C44^WT^ | QC1 | 2 | 2 | 2 | 0 | 2 | 4 |
|  | QC2 | 0 | 0 | 1 | 2 | 4 | 6 |
|  | QC3 | 0 | 1 | 1 | 5 | 2 | 2 |
| C44^SNP^ | QC1 | 3 | 0 | 3 | 5 | 6 | 4 |
|  | QC2 | 4 | 2 | 1 | 4 | 2 | 5 |
|  | QC3 | 5 | 4 | 2 | 6 | 5 | 3 |
| C45^WT^ | QC1 | 3 | 2 | 6 | 2 | 6 | 5 |
|  | QC2 | 3 | 0 | 0 | 7 | 3 | 5 |
|  | QC3 | 5 | 4 | 3 | 7 | 7 | 3 |
| C45^SNP^ | QC1 | 3 | 2 | 2 | 2 | 5 | 4 |
|  | QC2 | 1 | 3 | 1 | 6 | 6 | 4 |
|  | QC3 | 5 | 3 | 3 | 7 | 3 | 1 |

| **b** |  | **freeze/ thaw stability** | |
| --- | --- | --- | --- |
|  |  | **accuracy** (mean of n=3) | **deviation from freshly processed QCs** (mean of n=3) |
| C22^WT^ | QC2 | 104 | 2% |
|  | QC3 | 99 | -2% |
| C22^SNP^ | QC2 | 103 | 2% |
|  | QC3 | 97 | 0% |
| C36^WT^ | QC2 | 106 | 4% |
|  | QC3 | 98 | -1% |
| C36^SNP^ | QC2 | 104 | 2% |
|  | QC3 | 99 | -2% |
| C37^WT^ | QC2 | 114 | 9% |
|  | QC3 | 103 | 1% |
| C37^SNP^ | QC2 | 104 | 0% |
|  | QC3 | 98 | -1% |
| C39^WT^ | QC2 | 107 | 8% |
|  | QC3 | 101 | 2% |
| C39^SNP^ | QC2 | 105 | -1% |
|  | QC3 | 102 | -2% |
| C40^WT^ | QC2 | 107 | 5% |
|  | QC3 | 99 | -4% |
| C40^SNP^ | QC2 | 103 | -3% |
|  | QC3 | 100 | -3% |
| C42^WT^ | QC2 | 104 | 2% |
|  | QC3 | 100 | -4% |
| C42^SNP^ | QC2 | 105 | 2% |
|  | QC3 | 100 | -4% |
| C43^WT^ | QC2 | 103 | -2% |
|  | QC3 | 108 | -2% |
| C43^SNP^ | QC2 | 105 | -4% |
|  | QC3 | 107 | -3% |
| C44^WT^ | QC2 | 110 | -4% |
|  | QC3 | 115 | -1% |
| C44^SNP^ | QC2 | 109 | -6% |
|  | QC3 | 115 | -2% |
| C45^WT^ | QC2 | 108 | -6% |
|  | QC3 | 117 | -2% |
| C45^SNP^ | QC2 | 109 | -3% |
|  | QC3 | 112 | -2% |

| **c** |  | **benchtop stability** | |
| --- | --- | --- | --- |
|  |  | **accuracy** (mean of n=3) | **deviation from freshly processed QCs** (mean of n=3) |
| C22^WT^ | QC2 | 103 | 1% |
|  | QC3 | 102 | 1% |
| C22^SNP^ | QC2 | 104 | 2% |
|  | QC3 | 100 | 3% |
| C36^WT^ | QC2 | 106 | 4% |
|  | QC3 | 98 | -1% |
| C36^SNP^ | QC2 | 101 | -1% |
|  | QC3 | 98 | -2% |
| C37^WT^ | QC2 | 106 | 1% |
|  | QC3 | 101 | -1% |
| C37^SNP^ | QC2 | 103 | 0% |
|  | QC3 | 99 | -1% |
| C39^WT^ | QC2 | 106 | 8% |
|  | QC3 | 98 | -1% |
| C39^SNP^ | QC2 | 102 | -4% |
|  | QC3 | 100 | -4% |
| C40^WT^ | QC2 | 109 | 6% |
|  | QC3 | 101 | -3% |
| C40^SNP^ | QC2 | 104 | -1% |
|  | QC3 | 101 | -2% |
| C42^WT^ | QC2 | 102 | 1% |
|  | QC3 | 101 | -3% |
| C42^SNP^ | QC2 | 104 | 1% |
|  | QC3 | 101 | -3% |
| C43^WT^ | QC2 | 110 | 4% |
|  | QC3 | 109 | -1% |
| C43^SNP^ | QC2 | 109 | 1% |
|  | QC3 | 107 | -3% |
| C44^WT^ | QC2 | 117 | 1% |
|  | QC3 | 114 | -2% |
| C44^SNP^ | QC2 | 114 | -2% |
|  | QC3 | 115 | -2% |
| C45^WT^ | QC2 | 116 | 1% |
|  | QC3 | 117 | -3% |
| C45^SNP^ | QC2 | 114 | 1% |
|  | QC3 | 112 | -2% |
